# Principles for Good Practice in the Conduct of Non-interventional Studies: The View of Industry Researchers

**DOI:** 10.1007/s43441-023-00544-y

**Published:** 2023-07-17

**Authors:** Virginia Acha, Bart Barefoot, Ariadna Juarez Garcia, Valerie Lehner, Raffaella Monno, Susan Sandler, Almath Spooner, Patrice Verpillat

**Affiliations:** 1 MSD, 2 Pancras Square, London, UK; 2 GSK, London, UK; 3 BMS, London, UK; 4 Servier, Suresnes, Île-de-France, France; 5 Chiesi, Parma, Italy; 6 Janssen-Cilag Limited, High Wycombe, UK; 7 AbbVie, Dublin, Ireland; 8 Merck Healthcare KGaA, Darmstadt, Germany

**Keywords:** Non-interventional studies, NIS, Good practice, Hypothesis evaluating treatment effect, HETE, Real-world evidence, RWE, Real world data, RWD

## Abstract

This reflection paper presents a consolidated view of EFPIA on the need for principles for good practice in the generation and use of non-interventional studies (NIS), including overarching principles such as the registration of hypothesis evaluating treatment effect (HETE) studies. We first define NIS and the important adjacencies to clinical trials and relationship with real-world evidence (RWE). We then outline the principles for good practice with respect to appropriate research design, study protocol, fit-for-purpose variables and data quality, analytical methods, bias reduction, transparency in conduct and use, privacy management and ethics review. We conclude with recommendations for action for the research community to promote trust and credibility in the use of NIS.

## Introduction

Evidence generation for medicines, such as drugs, biologics and/or vaccines, includes a range of approaches. Although clinical trials (CTs) remain the research modality most required and relied upon by health authorities for establishing clinical safety and efficacy, the lifecycle of evidence generation for medicines increasingly includes non-interventional studies as a valuable component for assessment both in pre- and post-licensure settings [1, 2]. CTs are defined in law and in technical standards, through an established body of rules and norms incorporated in Good Clinical Practice ([3]: (p. 2), [4]). NIS are less well defined (although many global regulatory efforts are underway [5, 6]), sometimes are described as what they are not (as defined in the EU Clinical Trials Regulation as a “clinical study other than a clinical trial” [6]) and also referred to by different terms (e.g., observational studies by the US Food and Drug Administration [7]. Both CTs and NIS are valuable means to generate evidence; their value in a particular case depends on their fitness to address the research question posed. Both methods require robust research practices, even if there is more established and comprehensive guidance for CTs than for NIS.

As part of the growing trend and need for NIS, the research-based pharmaceutical industry has called [8] for clear principles from regulatory authorities for data quality and interoperability, access, analysis and regulatory acceptance to enable the greater use of data to assess patient outcomes in real-world settings. In this reflection paper authored by an expert group of sponsors and users of NIS, we likewise recognize that this expanded use of real world data (RWD) requires greater clarity on data quality standards, the measures to establish interoperability across data sources and the need for access to the data under the principles of Findability, Accessibility, Interoperability and Reusability (FAIR) [9]. We also call for international dialogue and alignment amongst the research community on suitable study methods and conduct and availability of the data; that is, there is a need to set out principles for good practice that should define the use and conduct of NIS. Our emphasis here is on the conduct of NIS for the purpose of further understanding treatment effects (e.g., causal inference), and not NIS purely focused on disease etiology (e.g., disease registries). Moreover, we recognize that such research not only must incorporate the scientific principles which we will outline, but also ethical and governance aspects to protect individuals’ rights in the confidentiality, security, stewardship, and appropriate use of data.

The paper is organized as follows. We review the key issues for NIS from the research-based pharmaceutical industry perspective and our aims to contribute to the principles for good practice discussion. We describe the scope of NIS for which these practices should apply, and then set them out in turn. We conclude with a call for action to the research community to ensure that these proposed principles for good practice do not remain on paper but are implemented for the benefit of health research and the patients who depend on progress.

## Non-interventional Studies

NIS play an increasing role in the generation of evidence to define and demonstrate the value of medicines and vaccines. The variety in research objectives, study designs, data and methods presents a challenge for standardization. Likewise, regulators and other decision-makers across the globe evaluating evidence generated from NIS hold different expectations regarding their use and fitness for purpose. This diversity within and across regions, including across and within stakeholder groups, persists still, although efforts are underway to build consensus where possible. Although regulatory guidance for NIS has been established in the post-licensing evidence generation setting [10, 11], these expectations and their formalization into regulatory guidance for both pre- and post-licensing use have been evolving through policy programmes and experience. The International Council for Harmonisation of Technical Requirements for Pharmaceuticals for Human Use (ICH) has recently concluded a revision of technical guidance E8 “General Considerations for Clinical Studies” that addresses both interventional and NIS (referred to as ‘observational studies’) [12], and in April 2022 the ICH Management Committee endorsed the establishment of a new ICH guideline on “General principles on plan, design, and analysis of pharmacoepidemiological studies that utilize real-world data for safety assessment of medicines” [13]. In 2021 alone, several guidance documents on real-world evidence (RWE) [14]–draft and final–were published by the US Food and Drug Administration (FDA) [5, 15], European Medicines Agency, (EMA) [16], UK Medicines and Healthcare Products Agency (MHRA) [17, 18], Chinese Centre for Drug Evaluation (CDE) [19] of the National Medical Products Administration (NMPA), Taiwanese Food and Drug Administration (TFDA) [20], Australian Therapeutic Goods Administration (TGA) [21] and Japanese Pharmaceuticals and Medical Devices Agency (PMDA) [22]. Recent FDA draft guidance ([5]: p. 2) has provided a helpful definition for NIS that identifies the key dimensions of this evidence generation method, although it is directed towards studies performed with a marketed drug. NIS can have other applications, and so for the purposes of this paper, we will define NIS as:

a *non-interventional study* (also referred to as an observational study) is a type of study in which all participants receive routine clinical care and are not assigned per protocol to a specific treatment or health intervention. Data from these studies are often evaluated using epidemiological methods [23]. They can include a systematic assessment of events without interfering with their course. Retrospective studies are non-interventional. Prospective studies may be non-interventional if the treatment choices and health interventions for an individual are according to clinical practice and have no bearing on their inclusion in the study. However, the exact definition and scope varies across regulatory and legal jurisdictions. Non-interventional studies can use both primary and secondary data.

NIS include many types of studies, i.e., including case–control studies and observational cohort studies, using data collected through primary and/or secondary (e.g., health records or claims-based analyses) means. As this definition indicates, NIS differ from clinical trials in that the study design does not affect treatment choice or health intervention for the patient (i.e., non-interventional). Patients are therefore not assigned based on a protocol for treatment and any non-routine care. Additional diagnostic or monitoring procedures can be applied to the patients, and epidemiological methods are used in the study design and for the analysis of collected data. From a data capture perspective, NIS can include both primary collection of data (i.e., data that are collected specifically for the purpose of the study in question) as well as secondary data use. In the case of secondary data use, data have often been collected for purposes other than research, and so the research team faces greater challenges regarding the quality and fitness of the data to address the research questions [24]. Of course, these data also provide opportunities to link multiple data sources at the patient level and thereby potentially enhance the rigor and richness of the study.

As with so many areas of science, the COVID-19 pandemic has highlighted the need to embrace the totality of evidence to expedite our epidemiological understanding of disease and the value of existing and new preventive and therapeutic interventions. There is a general recognition that NIS will have an important role to play in completing the totality of the evidence required for novel vaccines and treatments as well as label expansions for existing therapies [25, 26].

The strength of the evidence produced by NIS relies upon the application of defined good science principles and practices. Building on previous initiatives, notably the Good Pharmacoepidemiology Practices (GPP) issued by ISPE [27], contributions from FasterCures [28] and patient registries and registry-based studies sponsored by patient organizations and pharmaceutical companies, the Joint Task Force [29] of the International Society for Pharmacoeconomics and Outcomes Research (ISPOR) and the International Society for Pharmacoepidemiology (ISPE) set forth recommendations regarding good procedural practices in order to enhance confidence in the evidence derived from NIS. The recent FDA [5] and MHRA guidances [17, 18] also outline general principles with regard to sponsor responsibilities to sustain good science.

To contribute to the growing norms and standards for NIS conduct and to help the research community as a whole to achieve ‘good science’ in action, we–as representatives of sponsors of NIS-set out the following *overarching principles for good practice in NIS*.

### Scope for the Good Practice Principles

The overarching Good Science Principles discussed below are applicable to any NIS. However, as we believe the norms and standards for NIS research practices will take time to develop, we propose to prioritize adherence of these principles for those NIS that are conducted to assess safety and effectiveness of medicines and vaccines with the intention to be used to support regulatory, health technology assessment (HTA) or payer decision-making.

Specifically, our priority focus for Good Science Principles includes the NIS which can be described as hypothesis-evaluating studies to assess medicine/vaccine safety or effectiveness (defined as Hypothesis Evaluating Treatment Effect or HETE studies) to inform decision-making by regulatory agencies, HTA bodies and payers. The HETE terminology was defined by the ISPOR-ISPE Special Task Force on real-world evidence in healthcare decision making as:

[““[t]hese studies evaluate the presence or absence of a prespecified effect and/or its magnitude. The purpose of a HETE study is to test a specific hypothesis in a specific population. When evaluated in conjunction with other evidence, the results may lead to treatment recommendations by providing insights into, for example, whether a treatment effect observed in RCTs gives the same result in the real world where low adherence and other factors alter treatment effectiveness” [29].

For clarity, the treatment effect to be assessed in HETE NIS encompasses both safety and effectiveness outcomes for medicines and vaccines.

### Overarching Principles

We believe that for the successful conduct of HETE research for regulatory, HTA and payer decision-making purposes, the following Good Science Principles should be employed.

#### Appropriate Research Design for the Research Question Considered

The choice of research design must follow the nature and intent of the research question under consideration. The research intent and rationale should be a priori clarified as either exploratory/hypothesis generating or hypothesis testing/confirming (through validating or replicating results in multiple study designs or populations). These different objectives often frame different stages of scientific progress on a given topic and the expectations for the study results.

The study design should be developed to answer the research question directly and comprehensively as possible to confidently confirm or reject the hypothesis, and to minimize the introduction of bias and quantifying residual bias. There is considerable scholarship already that addresses best practices in study design and methods [30, 31], as well as reflections on the practical implementation of these [32], reflecting the central importance of research design and planning. Clearly communicating this design and approach is equally important, and greater use of diagrams and study design visualization can improve transparency. The ICH Guideline E9 (R1) *Statistical Principles for Clinical Trials: Addendum: Estimands and Sensitivity Analysis in Clinical Trials *[33] provides a structured framework to formulate clinical trial objectives, as well as the design, conduct, analysis and interpretation of clinical studies, including NIS.

#### The Study Protocol is the Cornerstone of the Research Process

The study protocol (and the statistical analysis plan (SAP)), which outlines the design of the study, provides the translation of the research design into practice, and thus the value of the study depends upon the accuracy of this translation into a robust research plan. However, a good research design does not automatically result in a good protocol; the practical features of the research context can be challenging, and decisions made in the translation from proposed study design to study execution need to be documented and explained so that the study and its outcomes can be well assessed, interpretable and, where appropriate, replicated. Moreover, the protocol must be finalized *before* a study commences. Guidance on developing appropriate study protocols is readily available, e.g. the foundational guide provided by ENCePP [34] and from EMA [10], and should be reviewed by those performing non-interventional research. Recently, a public–private collaboration developed a study implementation template –STaRT-RWE [24]–that aims to translate the key study parameters identified by the ISPOR-ISPE joint task force. The template [35] outlines details to capture regarding administrative information, study design, study population, study parameters and measures, providing not only a means to document the study, but to also assist the research team to apply Good Science Principles. Of course, templates are only research tools, the choice and application of which may require flexibility depending on the specifics of the study. Moreover, some studies may be subject to additional protocol requirements depending upon the purpose. The protocol can facilitate engagement with RWD controllers and processors [36] with respect to terms of engagement, methodological alignment and contractual approval.

The protocol needs to include well-defined population selection criteria, definition of key variables (such as exposures, covariates, as well as outcome measures that align with the research objectives (i.e., hypotheses explored, use of validated algorithms and/or endpoints); and the data source(s), including the way data have been or will be collected, need to be carefully selected and detailed in the protocol. The study sponsor will undertake a feasibility assessment [37], which can be critical to ensuring a fit-for-purpose evaluation (which can go beyond quality and to the estimates of study power and precision) of the data source(s) selected in close alignment with the protocol.

Where there are limitations in the data available (e.g., missing data) in meeting the research objectives, the approach taken should be explained and documented within the protocol and the SAP, and ultimately in the study report. The data collected must be appropriate to address the research question posed and sufficient to reach substantiated findings and conclusions. The data must also be accessible under terms that allow the study to be replicated by third parties if possible or required (e.g., regulatory audit).

#### ‘Fit-for-purpose’ Variables and Data Quality

The value of the study will be strongly determined by its design, including the relevant variables needed to address the research question posed. Stakeholders should be consulted to determine the variables of interest, taking into consideration the purpose of study and novelty of approach.

Once variables are determined, the availability of “fit-for-purpose” data is the next step. Access to good quality data for use in NIS remains an issue globally, beginning with the challenge of aligning on data quality terms and standards [38–40]. For studies involving primary collection, effective access to data for study is limited by the ability to generate data, following appropriate primary data collection standards [41]. For secondary use of data, these limitations include the availability, quality, heterogeneity, relevance, and terms of use of health data. These challenges are made more complex by the distribution of data across multiple repositories compounded by varying interoperability standards, quality, structures, and governance policies, including respect for all privacy considerations. And yet, conducting NIS fundamentally requires access to the requisite data, much of which will be held in public health repositories but also increasingly by private data providers. Some data will also be captured and held by the individual, through health apps, mobile devices, and monitors. As such, any progress in the use of NIS will depend upon the availability of appropriate and functional arrangements with this variety of data sources and owners for the use of data.

The quality of data needs to be sufficiently validated and reliable to address the research questions. Assessing and managing data quality represents the majority of data curation activities prior to data analysis, ideally involving a quality audit and feedback loop. The data used and the related pre-processing of such data need to be well described in the research protocol, identifying known and potential limitations to allow for the appropriate result interpretation and response.

#### Validated and Well Described Analytical Methods

Besides ensuring that the data are appropriate and of sufficient quality, the analytical methods applied to interrogate the data must also be fit-for-purpose and verifiable. All research should be reproducible in principle, and so the analytical methods and tests should be well described, including details of any deviations from the original analysis plan that have taken place. Increasingly advanced analytics, such as using artificial intelligence/machine learning methods, are being utilized to analyze data, and besides detailing these in the protocol, these should also be specified in the SAP when applicable. The sponsor needs to determine a priori whether the methods will be adequate to meet the needs of the research before the finalisation of the SAP, and there should be regulatory guidance to support the sponsor’s decision [42]. During the initial data analysis, researchers may identify data relationships that may be material to the study and could trigger additional non pre-specified analyses. Results of this would need to be clearly described in the study report as an a posteriori finding (18: (3.8)). However, these a posteriori* elaboration* should be avoided where possible for HETE NIS, which should keep to the pre-specified hypotheses tests.

#### Reducing Bias Including Confounding [43]

Supporting any evidence-based decision-making with RWE requires transparent and reproducible scientific study planning and adherence to pharmacoepidemiological and statistical principles [27] to minimize bias including confounding [44]. The absence of randomization in NIS raises concerns about risk of bias particularly selection bias and confounding. While these are legitimate and important concerns, it should be noted that randomization is not the only suitable method to reduce bias, as NIS can generate RWE that can support decision-making under defined conditions [45]. There is increasing consensus regarding methodological standards and good procedural practices for NIS comparing the effectiveness and safety of treatments, and there are multiple methods capable of reducing bias such that these studies may support valid causal inferences [46]. Potential sources of bias and confounding, and mitigation strategies to address these should be considered in the analytical planning (SAP) and discussed in any reporting of study results.

#### Transparency for the Conduct and Use of Non-interventional Studies

Research transparency is important for good science and provides access to regulators, HTAs/payers, the medical community, and patients to the information they need to make informed decisions about healthcare. Transparency in study conduct is especially important to support the credibility of NIS. While transparency by itself is no guarantee of study quality or acceptability, registration, data traceability and clear reporting of studies can instill greater confidence in end-to-end research processes and mitigate the risk of publication bias and other biases [23, 47]. This includes also being able to describe the provenance of data, which underscores the need for discussion about the appropriate transparency of the processes, SOPs, manipulations, quality assurance and quality controls of the data provider/ data. These transparency measures are valued not only by the research community but are increasingly expected for scientific publication [48] and encouraged as per the Principles 35 and 36 of the World Medical Association (WMA) Declaration of Helsinki [49].

However, for NIS to be widely accepted, we need to build trust in the research and its outcomes through establishing the provenance of the research,^1^ and not simply transparency of the data used. Provenance is about establishing the trust, credibility, and reproducibility of the research by allowing the reader to understand the decisions taken by the research team in defining the study design, data provenance, data and variables, and the research methods and analysis undertaken to arrive at the results, which are then shared. Whilst this reflects established good science practices, what is needed is global alignment on the practical steps and expectations sponsors must undertake to establish this study provenance. Initiatives underway through professional societies (ISPE, ISPOR) recognize the need for good practice guidance to support provenance, and the FDA explicitly set out its expectations in recent draft guidance on “*Transparency Regarding Data Collection and Analysis*”, but there are areas still to clarify and agree on ([5]: p. 4). As representatives of NIS sponsors, we *set out for consideration* the following steps to provide the basis for an aligned approach to establish study provenance:

- Where not otherwise legally required, this practice should focus on NIS that are comparative hypothesis evaluating treatment effect (HETE) studies to support regulatory, HTA and/or payer decision-making. We align with the conceptual approach that ISPOR/ISPE ([29], p. 1004) [23] take with respect to study registration. Registration of HETE studies that support regulatory, HTA and payer decision-making should be voluntary and encouraged. Use of incentives to register could be a valuable tool to encourage participation from all sponsors, supported by guidance and multistakeholder dialogue to support good research practices.
- The *timing of disclosure of relevant study and result details must not prejudice the rights of the sponsor and study partners*. Where premature disclosure of such details could harm the study and/or the rights of the sponsor, proposed mechanisms such as a ‘locked box’ [29, 50], or other acceptable method of deferral and redaction of commercially confidential information would sustain the scientific rigor of the research and protect the rights of the sponsor and study partners.
- When pursued, *registration of HETE studies should occur after* protocol finalisation and precede initiation of the study. *Registration should include relevant details of the study protocol* or reflect the current guidance and practice of established study registers like the European Union electronic register of Post-Authorisation Studies (EU-PAS) or Clinicaltrials.gov registers.
- Current practice anticipates what would be provided in the scientific publication i.e., the relevant details *of the study protocol*. These elements would be included in registration, with exception of elements where early disclosure would prejudice the rights of the sponsor and study partners.
- Global coordination is necessary *to avoid the proliferation of several of such study registers,* which may fragment the data captured and could lead to burdens of duplication and confusion. There are already considerable challenges and efforts [51] underway to map existing real world data sources; every effort should be made to align internationally on an approach that resolves the reporting terms and sites from the outset. To support more effective research and avoid duplication of records and resources, we should encourage cross-referencing (even mutual recognition) among registers.
- An appropriate registration process would support efficient registration of NIS. For this reason, making use of an existing central study register, such as the EU-PAS register or Clinicaltrials.gov is an option. Where applicable in broader registers, NIS should be clearly differentiated from interventional trials.
- Disclosure of study results should follow established scientific practice, that is *publication of study results (irrespective of the direction and strength of the study findings) in peer-reviewed scientific journals,* to allow for the validation of findings and advance the broader research agenda. However, publication in such peer-reviewed journals is not the final decision of the study authors, and so when and where publication is not achieved, disclosure of study results will at least be available via the study register and potential other sites (e.g. sponsor websites) [52]. Once available through the scientific literature, authors can then publicly address any methodological criticisms of the study that may arise.

#### Privacy Requirements

NIS must be conducted in accordance with the privacy requirements for the jurisdiction of relevance for the data, and these vary globally. This can be challenging where relying on data from multiple jurisdictions in an NIS. Moreover, data privacy law and expectations are developing at different rates across countries and regions. NIS must be *compliant with laws and regulations affecting privacy rights* (e.g., EU GDPR) *and follow privacy by design principles *[53]. We need harmonized guidance developed in collaboration between regulatory authorities, data protection authorities and industry to support NIS. We thus welcome the intention of the EMA to develop a Q&A document [54] for this purpose, which will be a valuable resource. Support will also be needed to enable sponsors and custodians to collaborate in building secure data-sharing environments foreseen in emerging policy guidance.

Data protection should be embedded in the governance of NIS from the planning stages of the study, thereby achieving data protection by design. Research teams should involve data protection expertise and advice throughout the conduct of the NIS.

#### Ethics Review of NIS

Non-interventional studies must be reviewed and approved by Ethics Committees in line with local regulations and law [52]. Approval by a Research Ethics Committee is often required for the study to proceed, depending on local regulations. Others [55] have noted the considerable variability in approach and requirements for ethical review in Europe and likely in other regions. All stakeholders would benefit from sharing experience and identifying best practices in ethics reviews for NIS conducted to date, and harmonization of requirements for Ethics Reviews would be very valuable.

## Conclusion

### Call for Action

We recognize and welcome international developments by regulators to advance the use of RWD/RWE for healthcare, including the FDA’s Advancing Real-World Evidence Program [56] and the 10 priority recommendations from the HMA EMA Joint Big Data Task Force which would foster robust data environment and research practices [57]. As developers of innovative therapeutics and vaccines, we join this effort and call on the international research, regulatory, HTA and payer community to work with us to (see Table 1):

- Agree on a common set of *Good Practice Principles* for NIS, building on existing related guidelines [27, 58] in order to establish comprehensive guidance to support the effective conduct of NIS and increase confidence in using these studies for healthcare decision making,
- Ensure that NIS that are used to assess treatment safety and effectiveness (HETE studies) adhere to these Good Science Principles for the benefit of patients and public health, and
- Commit to building robust quality data that can enable meaningful NIS.

**Table 1:** Three key steps to good practice principles

1	Common set of principles for Good Practices for NIS	Globally agreed, built on existing guidelines
		Comprehensive guidance to support NIS researchers
		Increase confidence for decision makers
2	Focus on NIS HETE studies	Ensure NIS HETE Studies used for decision-making adhere to these Good Practice Principles
3	Build trust through building quality data	Commit to building robust quality data that can enable meaningful NIS and evidence

## Footnotes

1 We note that the term ‘provenance’ typically refers to the data only. We are purposefully introducing the use of this term with respect to the study itself, to provide the analogy to confirm the importance of understanding not only what is being assessed (the data), but how (the study).

## Definitions

Hypothesis-generating/exploratory studies: Studies that seek relationships and patterns in a specified dataset or related data sets. These relationships and patterns can be tested in subsequent, appropriately designed (HETE) studies, using primary or secondary data [23].
Hypothesis evaluating treatment effect (HETE) studies: Comparative effectiveness, comparative safety, or causal inference studies. Studies that evaluate the presence or absence of a prespecified effect and/or its magnitude. “Effect” in this usage includes both effectiveness and safety. HETE studies test a hypothesis in a specific population. When evaluated in conjunction with other evidence, the results may lead to treatment recommendations. For example, HETE studies might provide insights into whether a treatment effect observed in randomized controlled trials is the same in the real world, where low adherence rates and other factors could alter treatment effectiveness. HETE studies can use primary or secondary data [23]. Although the concept has been developed to assess treatments, this should be expanded to consider vaccines (and other prevention measures) effectiveness and duration of protection. Please note that in some instances, HETE has been defined as hypothesis evaluating treatment *effectiveness,* which would exclude safety studies. We support the definition of HETE as described here (hypothesis evaluating treatment effect, also including safety).
Interventional studies: (Also referred to as ‘clinical trials’) Studies in which participants are assigned to a study intervention, standard of care, treatment that is not standard of care or placebo to measure the impact of intervention [23]. Randomization in assignment is frequently used in these studies, and in that case, these are referred to as RCTs.
Non-interventional studies: Studies in which participants receive routine clinical care and are not assigned per protocol to a specific treatment or health intervention. Data from these studies are often evaluated using epidemiological methods [23]. They can include a systematic assessment of events without interfering with their course. Retrospective studies are non-interventional. Prospective studies may be non-interventional if the treatment choices for an individual are according to clinical practice and have no bearing on their inclusion in the study. However, the exact definition and scope varies across regulatory and legal jurisdictions [59]. Non-interventional studies can use both primary and secondary data. The European Commission summarizes the definition of a non-interventional study as “a clinical study other than a clinical trial” [6]. More recently, the European Medicines Agency has published a decision tree to determine whether a clinical study is a clinical trial or a non-interventional study [59] that pivots on the assignment of patients and their care and the deviation from standard of care. The U.S. FDA defines ‘observational studies’ as ‘non-interventional clinical study designs that are not considered clinical trials’ [7] The FDA defines NIS as “a type of study in which patients received the marketed drug of interest during routine 67 medical practice and are not assigned to an intervention according to a protocol [5].
Publication bias: The failure to publish the results of a study “on the basis of the direction or strength of the study findings”. This failure to publish introduces a bias, which impacts the ability to accurately synthesize and describe the evidence in a given area [23].
